# Adaptation costs for climate change-related cases of diarrhoeal disease, malnutrition, and malaria in 2030

**DOI:** 10.1186/1744-8603-4-9

**Published:** 2008-09-19

**Authors:** Kristie L Ebi

**Affiliations:** 1 ESS, LLC, Alexandria, VA 22304, USA

## Abstract

**Background:**

Climate change has begun to negatively affect human health, with larger burdens projected in the future as weather patterns continue to change. The climate change-related health consequences of diarrhoeal diseases, malnutrition, and malaria are projected to pose the largest risks to future populations. Limited work has been done to estimate the costs of adapting to these additional health burdens.

**Methods:**

The costs of treating diarrhoeal diseases, malnutrition (stunting and wasting only), and malaria in 2030 were estimated under three climate scenarios using (1) the current numbers of cases; (2) the projected relative risks of these diseases in 2030; and (3) current treatment costs. The analysis assumed that the number of annual cases and costs of treatment would remain constant. There was limited consideration of socioeconomic development.

**Results:**

Under a scenario assuming emissions reductions resulting in stabilization at 750 ppm CO_2 _equivalent in 2210, the costs of treating diarrhoeal diseases, malnutrition, and malaria in 2030 were estimated to be $4 to 12 billion. This is almost as much as current total annual overseas development assistance for health.

**Conclusion:**

The investment needs in the health sector to address climate-sensitive health outcomes are large. Additional human and financial resources will be needed to prevent and control the projected increased burden of health outcomes due to climate change.

## Background

The health impacts of climate change are diverse and wide-ranging. Weather and climate are among the factors that determine the geographic range and incidence of several major causes of ill health, including undernutrition, which affects 17% of the world's population in developing countries [1]; diarrhoeal diseases and other conditions due to unsafe water and lack of basic sanitation, which cause 2 million deaths annually, mostly in young children [2]; and malaria, which causes more than a million childhood deaths annually [3]. Table 1 provides the annual incidence of diarrhoeal disease, malnutrition, and malaria by WHO Region in 2002 [countries included in each region are provided in Additional file 1]. The numbers for malnutrition include only stunting and wasting, not all the health impacts, and do not include micronutrient deficiencies, such as of zinc and vitamin A, that also have serious health consequences.

**Table 1:** Annual incidence of diarrhoeal diseases, malnutrition (stunting and wasting) and malaria by WHO sub-region, 2002

**Sub-region**	**Population (000s)**	**Diarrhoeal diseases (000s)**	**Malnutrition (000s)**	**Malaria (000s)**	**Total (000s)**
Afr-D	301 878	389 842	5 033	180 368	575 243
Afr-E	353 598	449 192	5 912	176 651	631 755
Amr-A	328 176	77 578	137	0	77 715
Amr-B	437 142	390 590	1 124	2 866	394 580
Amr-D	72 649	73 271	603	718	74 592
Emr-B	141 835	96 324	585	363	97 272
Emr-D	351 256	345 605	4 523	16 898	367 026
Eur-A	412 512	79 219	134	0	79 353
Eur-B	219 983	78 509	649	0	79 158
Eur-C	241 683	47 886	262	0	47 912
Sear-B	297 525	179 213	2 251	6 951	188 415
Sear-D	1 262 285	1 051 538	18 040	21 568	1 091 146
Wpr-A	154 919	30 026	64	6	30 096
Wpr-B	1 546 770	1 225 188	7 035	1 838	1 234 061
World	6 122 211	4 513 981	46 352	408 227	4 968 560

Source: ; accessed 20 May 2007

The Fourth Assessment Report of the Intergovernmental Panel on Climate Change concluded that climate change has begun to negatively affect human health, and that projected climate change will increase the risks of climate-sensitive health outcomes [4]. The climate change-related health consequences of malnutrition, diarrhoeal diseases, and malaria are projected to pose large risks to future populations, particularly in low-income countries in tropical and sub-tropical regions.

The size of the projected impacts raises the question of how much it will cost to treat these additional cases of disease. To further the discussion of adaptation costs, this paper estimates of the costs of interventions to cope with additional cases of malnutrition, diarrhoeal diseases, and malaria due to climate change in 2030. The estimates are for the costs of climate change only. Population growth is not considered and there is limited consideration of socioeconomic development.

## Methods

The data sources used were (1) the current number of cases of diarrhoeal diseases, malnutrition, and malaria [; accessed 20 May 2007]; (2) the World Health Organization (WHO) Global Burden of Disease (GBD) study that projected the relative risks associated with climate change in 2030 for a range of climate-sensitive health determinants and outcomes [5]; and (3) published data on the costs of interventions for diarrhoeal diseases, malnutrition, and malaria, primarily from the project 'Disease Control Priorities in Developing Countries' . Assuming that the current annual number of cases of diarrhoeal diseases, malnutrition, and malaria would remain constant to 2030, the numbers of current cases were multiplied by the relative risks for climate change estimated by the Global Burden of Disease Study (under three different emission scenarios) to estimate the number of additional cases of these diseases that could be attributed to climate change in the year 2030. The numbers of additional cases were multiplied by the current costs of treatment per case to estimate the additional costs of treating climate change-related cases of diarrhoeal diseases, malnutrition, and malaria.

### WHO Global Burden of Disease study

The goals of the World Health Organization (WHO) Global Burden of Disease study were to produce the best possible evidence-based description of population health, the causes of lost health, and likely future trends in health in order to inform policy-making [6]. Twenty-six risk factors, including climate change, were assessed [5]. The GBD used two summary measures of population health, mortality and the Disability Adjusted Life Years lost (DALYs). DALYs provide a better measure than mortality of the population health impacts of diarrhoeal diseases, malnutrition, and malaria. The attributable burden of DALYs for a specific risk factor was determined by estimation of the burden of specific diseases related to the risk factor; estimation of the increase in risk for each disease per unit increase in exposure to the risk factor; and estimation of the current population distribution of exposure, or future distribution as estimated by modelling exposure scenarios. Counterfactual or alternative exposure scenarios to the current distribution of risk factors were created to explore distributional transitions towards a theoretical minimum level of exposure (e.g. for exposure to carcinogens, the theoretical minimum level of exposure would be no exposure).

For climate change, the questions addressed were what will be the total health impact caused by climate change between 2000 and 2030 and how much of this burden could be avoided by stabilizing greenhouse gas emissions [5]. The alternative exposure scenarios defined were:

• Unmitigated emission trends (i.e., approximately following the Intergovernmental Panel on Climate Change IS92a or business as usual scenario);

• Emissions reductions resulting in stabilization at 750 ppm CO_2 _equivalent by 2210 (s750); and

• Emissions reductions resulting in stabilization at 550 ppm CO_2 _equivalent by 2170 (s550).

Climate change projections were generated by the HadCM2 general circulation climate model [7]. The health outcomes included in the analysis were chosen based on sensitivity to climate variation, predicted future importance, and availability of quantitative global models (or feasibility of constructing them). The health outcomes selected were the direct impacts of heat and cold, episodes of diarrhoeal disease, cases of *Plasmodium falciparum *malaria, fatal unintentional injuries in coastal floods and inland floods/landslides, and non-availability of recommended daily calorie intake (as an indicator for the prevalence of malnutrition). Global and WHO specific region estimates were generated.

In the year 2000, the mortality attributable to climate change was estimated to be 154,000 (0.3%) deaths, and the attributable burden was 5.5 million (0.4%) DALYs, with approximately 50% of the burden due to malnutrition [5]. About 46% of the DALYs attributable to climate change were estimated to have occurred in the WHO South-East Asia Region, 23% in countries in the Africa region with high child mortality and very high adult male mortality, and 14% in countries in the Eastern Mediterranean region with high child and adult male mortality.

Additional files 2, 3, 4, provide the relative risk estimates for malnutrition, diarrhoeal diseases, and malaria, respectively, projected for 2030 under the alternative exposure scenarios [5]. Lower range relative risk estimates are not shown as they were 1.00 or close to 1.00.

For diarrhoeal diseases, developing countries were defined as those with per capita incomes less than US$6,000/year in 1990 US dollars [5]. For such countries, the exposure-response relationship used was a 5% increase in diarrhoeal incidence per °C increase in temperature. The study assumed that the climate sensitivity of diarrhoea would decrease with increasing GDP; once a country was projected to reach per capita incomes of UD$6,000/year (as estimated by EMF 14 [8]), then overall diarrhoea incidence was assumed to not respond to changes in temperature. The study assumed that diarrhoeal incidence in richer countries is insensitive to climate change. The relative risks for each region are a population-weighted average of the countries within the region.

For malnutrition, estimates of national food availability were based on the effects of temperature and precipitation, and the beneficial effects of higher CO_2 _levels, projected using the IBSNAT-ICASA dynamic crop growth models [9]. Principal characteristics of this model include no major changes in the political or economic context of world food trade or in food production technology; population growth follows the World Bank mid-range estimate (i.e. 10.7 billion by the 2080s); GDP accumulated as projected by EMF14 [8]; and a 50% trade liberalization in agriculture is introduced gradually by 2020.

Analyses suggested that the model output was positively related to more direct measures of malnutrition, including incidence of underweight, and stunting and wasting in children <5 years of age [5]. The relative risks of malnutrition in Additional file 3 were interpreted as being directly proportional to underweight; this applies to all diseases affected by underweight (including diarrhoea and malaria). The model output was used to generate mid-range estimates; the high relative risks were calculated as a doubling of the mid-range estimate.

**Table 2:** Projected excess incident cases of diarrhoeal diseases (000s) for alternative climate scenarios relative to baseline climate (mid and high estimates)

**Sub-region**	**Climate**	**2000**		**2030**	
		Mid	High	Mid	High

Afr-D	S550	3,898	11,695	19,492	38,984
	S750	7,797	15,594	23,391	50,679
	UE	7,797	19,492	27,289	62,375
Afr-E	S550	4,492	13,476	22,460	49,411
	S750	8,984	17,968	26,952	58,395
	UE	8,984	22,460	35,935	71,871
Amr-A	S550	0	1,552	0	4,655
	S750	0	1,552	0	4,655
	UE	0	1,552	0	6,206
Amr-B	S550	0	7,812	0	19,530
	S750	0	7,812	0	23,435
	UE	0	7,812	0	31,247
Amr-D	S550	733	2,198	1,465	5,129
	S750	1,465	2,198	1,465	5,862
	UE	1,465	2,931	1,465	7,327
Emr-B	S550	963	2,890	5,779	5,779
	S750	1,926	2,890	5,779	5,779
	UE	1,926	3,853	8,669	8,669
Emr-D	S550	6,912	10,368	0	41,472
	S750	6,912	10,368	0	44,929
	UE	10,368	17,280	0	65,665
Eur-A	S550	0	1,584	0	4,753
	S750	0	1,584	0	4,753
	UE	0	1,584	0	6,338
Eur-B	S550	785	2,355	785	5,496
	S750	785	2,355	785	6,281
	UE	785	2,355	785	7,066
Eur-C	S550	958	1,437	0	3,352
	S750	958	1,437	0	3,352
	UE	958	1,915	0	3,831
Sear-B	S550	1,792	3,584	0	8,960
	S750	1,792	5,375	0	10,753
	UE	3,584	7,169	0	14,337
Sear-D	S550	21,031	1.03	63,092	136,700
	S750	21,031	42,062	73,608	157,731
	UE	31,546	52,577	94,638	199,792
Wpr-A	S550	0	300	0	1,501
	S750	0	300	0	1,501
	UE	0	601	0	2,102
Wpr-B	S550	12,252	36,756	0	73,511
	S750	12,252	36,756	0	73,511
	UE	24,504	61,259	12,252	110,267

For malaria, estimates for the projected populations at risk of *Plasmodium falciparum *malaria were based on the MARA/ARMA model [5]. The model output was used to generate mid-range estimates; the high relative risks were calculated as a doubling of the mid-range estimate. Socioeconomic development was assumed to not affect the incidence of malaria.

## Results

### Estimated climate change-related excess incident cases of diarrhoeal diseases, malnutrition, and malaria in 2030

The total estimated excess incident cases of diarrhoeal diseases, malnutrition, and malaria in 2030 for the three scenarios (unmitigated emissions and stabilization at 550 and 750 ppm CO_2 _equivalent) are shown in Tables 2, 3 and 4. Given the current burden of these health outcomes and the relative risks from the Global Burden of Disease study, it is not surprising that the largest increases in climate change-attributable cases are projected to be in Africa and Southeast Asia. Table 5 compares current and projected (under the 750 ppm CO_2 _scenario) numbers of cases of diarrhoeal diseases, malnutrition, and malaria; climate change is projected to increase the numbers of cases by 3–10%. Smaller increases were projected under the lower emission scenarios.

**Table 3:** Projected excess incident cases of malnutrition (000s) for alternative climate scenarios relative to baseline climate (mid and high estimates)

**Sub-region**	**Climate**	**2000**		**2030**	
		Mid	High	Mid	High

Afr-D	S550	50	101	151	302
	S750	50	151	201	453
	UE	50	50	101	201
Afr-E	S550	59	118	177	355
	S750	59	118	236	473
	UE	59	59	118	296
Amr-A	S550	0	0	0	0
	S750	0	0	0	0
	UE	0	0	0	0
Amr-B	S550	22	34	56	112
	S750	34	79	134	247
	UE	0	0	0	0
Amr-D	S550	12	18	30	133
	S750	18	42	66	0
	UE	0	0	0	0
Emr-B	S550	6	12	18	35
	S750	12	23	35	76
	UE	0	0	0	0
Emr-D	S550	90	181	317	678
	S750	136	317	498	995
	UE	90	226	362	724
Eur-A	S550	0	0	0	0
	S750	0	0	0	0
	UE	0	0	0	0
Eur-B	S550	0	0	0	0
	S750	0	0	0	0
	UE	0	0	0	0
Eur-C	S550	0	0	0	0
	S750	0	0	0	0
	UE	0	0	0	0
Sear-B	S550	45	68	113	225
	S750	68	135	225	428
	UE	0	0	0	23
Sear-D	S550	722	1263	2165	4510
	S750	722	1804	3067	6314
	UE	902	1804	3067	5953
Wpr-A	S550	0	0	0	0
	S750	0	0	0	0
	UE	0	0	0	0
Wpr-B	S550	0	70	70	141
	S750	70	141	211	352
	UE	0	0	-70	0

**Table 4:** Projected excess incident cases of malaria (000s) for alternative climate scenarios relative to baseline climate (mid and high estimates)

**Sub-region**	**Climate**	**2000**		**2030**	
		Mid	High	Mid	High

Afr-D	S550	0	1804	1804	3607
	S750	0	1804	1804	5411
	UE	1804	1804	3607	9018
Afr-E	S550	3533	7066	12366	26498
	S750	3533	8833	15899	31797
	UE	7066	14132	24731	49462
Amr-A	S550	0	0	0	0
	S750	0	0	0	0
	UE	0	0	0	0
Amr-B	S550	57	115	229	459
	S750	86	143	287	545
	UE	115	258	430	860
Amr-D	S550	7	14	29	65
	S750	7	22	36	72
	UE	14	29	57	122
Emr-B	S550	0	0	0	0
	S750	0	0	0	0
	UE	0	0	0	0
Emr-D	S550	607	1183	2535	5069
	S750	676	1352	3211	6252
	UE	1014	2197	4900	9970
Eur-A	S550	0	0	0	0
	S750	0	0	0	0
	UE	0	0	0	0
Eur-B	S550	0	0	0	0
	S750	0	0	0	0
	UE	0	0	0	0
Eur-C	S550	0	0	0	0
	S750	0	0	0	0
	UE	0	0	0	0
Sear-B	S550	0	0	0	0
	S750	0	0	0	0
	UE	0	0	0	0
Sear-D	S550	0	0	0	70
	S750	0	0	70	70
	UE	0	0	70	139
Wpr-A	S550	0.4	0.8	1.5	3
	S750	0.5	1.0	2	4
	UE	0.8	1.6	3	6
Wpr-B	S550	110	221	404	790
	S750	147	276	478	974
	UE	221	441	772	1526

**Table 5:** Comparison of current diarrhoeal disease, malnutrition, and malaria cases with estimated additional cases due to climate change in 2030 assuming the 750 ppm of CO_2 _scenario (thousands of cases)

	**Diarrhoeal diseases**	**Malnutrition**	**Malaria**
Current cases	4,513,981	46,352	408,227
Climate change attributable cases in 2030	131,980	4,673	21,787
% increase	3%	10%	5%

### Annual costs of interventions for diarrhoeal diseases, malnutrition, and malaria

Annual costs of intervention for diarrhoeal diseases, malnutrition, and malaria  were based on currently deployed interventions and did not include costs of implementing programs (including infrastructure and health care personnel costs) in new areas if these diseases increase their geographic range, as is projected. The costs of initiating programs in new areas can be significant, and include costs of infrastructure (i.e. building clinics, costs for equipment and drugs), training new personnel, maintenance costs, etc. Excluding the costs of implementing programs that are currently being scaled up across Africa with the help of the Global Fund, the US President's Malaria Initiative, and others, substantially underestimates the cost of controlling malaria.

There are three major diarrhoea syndromes requiring treatment: acute watery diarrhoea that results in varying degrees of dehydration; persistent diarrhoea that last 14 days or longer, manifested by malabsorption, nutrient losses, and wasting; and bloody diarrhoea caused by inflammation of the intestinal tract. Viruses, bacteria, protozoa, and helminthes can cause diarrhoea. Diarrhoeal diseases affect all populations, with the largest health burdens among the poor. The costs of two sets of intervention for treating diarrhoeal diseases in children under five were estimated: (1) breastfeeding promotion, rotavirus immunization, cholera immunization, and measles immunization; and (2) improvement of water supply and sanitation [10]. The average cost per child in 2001 US$ for (1) was $15.09 (the costs range from $0.71 per child for oral rehydration therapy in Indonesia to $104.30 per child for rotavirus immunization in South Africa) and for (2) was $53.00 ($25.00 for rural areas and $81.00 for urban areas).

The average costs of nutritional interventions per child for addressing underweight range from $17.40 to $23.09, and include breastfeeding promotion, child survival programs (with a nutritional component), nutritional programs, and growth monitoring and counselling [11]. These costs are very conservative; Edejer et al. [12] estimated the annual per capita cost of providing food to improve child health in Africa D and SEAR-D was $int (international dollar) 116.23, and the cost per recipient was $int 310.91 to 317.30. An international dollar is a hypothetical unit of currency that has the same purchasing power that the US$ has in the US at a given point in time, thus showing the average value of local currency units within each region's borders. Using these estimates would increase the estimated costs by more than 10-fold.

The costs of two sets of interventions for malaria were estimated: (1) insecticide-treated bednets plus case management with artemisinin-based combination therapy plus intermittent presumptive treatment in pregnancy; and (2) indoor residual spraying plus (1) [13]. The average cost for (1) for Africa D and E was $int 88.50 and the average cost for (2) was $int 123.5; these are incremental costs per disability adjusted life year lost and did not include the costs of implementing new malaria control programs. These cost estimates are not on the same basis as those for diarrhoeal diseases and malnutrition (which were for the cost of treatment intervention per child); however, no adjustments were made in the analysis.

Table 6 summarizes the projected excess costs in millions of US$ in 2030 to manage the excess cases of diarrhoeal diseases, malnutrition, and malaria due to climate change under the three scenarios. The total costs under S550 were estimated to be $3,333 to $10,689 million; the total costs under S750 were $3,992 to $12,603 million; and the total costs under UE were $5,852 to $17,957 million.

**Table 6:** Projected excess costs (million US$) in 2030 to manage climate change-related cases of diarrhoeal diseases, malnutrition, and malaria for three alternative climate scenarios relative to baseline climate (mid and high estimates)

**Scenario**	**Diarrhoeal Diseases**		**Malnutrition**		**Malaria**	
	Mid	High	Mid	High	Mid	High

S550	1,706	6,024	53.9 – 71.5	112.9 – 149.9	1,573 – 2,145	3,236 – 4,515
S750	1,983	6,814	81.3 – 107.9	162.5 – 215.6	1,928 – 2,691	3,994 – 5,573
UE	2,731	9,010	62.2 – 82.6	125.2 – 166.2	3,059 – 4,269	6,293 – 8,781

### Current health expenditures

Poor countries tend to have low health expenditures and to rely significantly on external donors [3]. Currently, there are a number of donors interested in investing in health, which is increasing overseas development assistance. Bilateral assistance for health rose from an annual average of US$ 2.2 billion during 1997–99 to US$ 2.9 billion in 2002 (Table 7) [14]. Within the UN system, development assistance rose from an annual average of US$ 1.6 billion during 1997–99 to US$ 2 billion in 2002. Commitments from the development banks remained stationary at about US$ 1.4 billion. However, changes in accounting at the World Bank to include financing for health-related activities in other sectors (i.e. water and sanitation, transportation, and social development), suggest that new commitments rose from about US$ 1 billion in 2001 to US$ 1.7 billion in 2003.

**Table 7:** Development assistance for health, selected years (millions US$)

**Source**	**Annual Average, 1997–1999**	**2002**
Bilateral agencies	2 560	2 875
Multilateral agencies	3 402	4649
European Commission	304	244
Global Fund to Fight AIDS, Tuberculosis, and Malaria	0	962
Bill & Melinda Gates Foundation	458	600
Total	6 724	9 330

Source: Hecht and Shah [14]

Therefore, for the 750 ppm CO_2 _scenario, the annual needs in 2030 would be almost as much as current total annual overseas development assistance for health. The estimate of investment needs does not account for socioeconomic changes, in particular increased population and income. Assuming the estimated costs of treatment per case do not differ between baseline cases and cases due to climate change, the total investment needs in 2030 for combating diarrhoeal disease would be $67 billion, malnutrition $2 billion, and malaria $36 to $50 billion.

## Discussion

Estimating the adaptation needs in the health sector is challenging. Most of the health outcomes that are projected to be affected by climate change are current problems; there will not be death certificates, hospital admissions, or records of visits to health care providers indicating that a particular event was due to climate change. Instead, as with some other environmental exposures (particularly indoor and outdoor air quality), models are used to estimate the proportion of a disease burden that can be attributed to climate change based on exposure-response relationships and projected changes in weather patterns. Uncertainties in models, from limited data through to inadequate specification of factors that influence the exposure-response relationship, will therefore lead to uncertainty as to the precise magnitude of the climate change impact.

The analysis makes a number of necessary, but unlikely assumptions, including that the number of annual cases of diarrhoeal disease, malaria, and malnutrition, and the cost of treatment would remain constant. Population growth is projected to increase under the medium variant from 6.1 billion in 2000 to 8.3 billion in 2030 [15]. Conducting a sensitivity analysis that incorporated these population increases would require assumptions of future incidence rates of these health outcomes, based on assumptions of socioeconomic development, including improvements in health care delivery, the rate of deployment of current interventions, and the development of more effective technologies. Using the current number of cases in the analysis in effect assumes that incidence will decrease as population increases, without attribution of the possible reasons for such a decline. If disease rates remain constant until 2030, then the number of cases due to climate change would increase.

Because of the large uncertainties, the costs estimated should be viewed only as indicators of the relative magnitude of health adaptation costs. Countries improve their public health and health care systems as they develop, which should decrease the burden of many climate-sensitive diseases. Costs of current treatments tend to decrease over time, although development of new, more effective treatments may cost more. However, there is an underlying assumption that currently developing countries will develop along similar pathways to those followed by the developed countries. There is ample evidence to suggest that the reality may be much more challenging. A key issue is water; most developing countries do not have as much available water as developed countries did when they were developing. Therefore, it will be more difficult to resolve issues such as access to safe water and sanitation. Also, malaria is much more difficult to control in Africa than it was in Europe and the US.

Another complexity is estimating the economic cost of injuries, illnesses, and deaths across multiple countries and regions. Issues include not just how to value a human life, but how to measure economically the life-course consequences of malnutrition, for example. Mortality is a commonly used metric, but is an inadequate measure of the affect of a health outcome on the family and on society; a death at age 80 and a death at age 2 would be counted equally while having different impacts. Similarly, malnutrition decreases learning ability, thus affecting lifelong earning potential, among a myriad of other impacts. Therefore, counting cases of disease also is insufficient for estimating total impacts.

Additional research could reduce some of the uncertainties in the analysis. The literature base underlying the exposure-response relationships is fairly thin; additional estimates in more regions would increase confidence in projected relative risks and would allow estimates of future climate change-attributable cases on smaller spatial scales. Additional research also is needed to better project how population growth, socioeconomic development, and other factors would likely influence future rates of climate-sensitive health determinants and outcomes. Development of a health model would facilitate both projections and identification where additional information would reduce uncertainty [16]. Linking such a model with integrated assessment models would take advantage of the their efforts to model population growth and economic development.

Bosello et al. [17] estimated the economic impacts of climate change in 2050 on temperature-related illnesses, diarrhoeal diseases, malaria, dengue fever, and schistosomiasis. Changes in morbidity and mortality were interpreted as changes in labour productivity and demand for health care. There was a mixed pattern of increases and decreases in GDP, welfare, and investment across world regions, with benefits estimated in high-income countries and losses primarily in low-income countries. The results showed that direct cost estimates, such as the present analysis, underestimate the full health costs (and benefits) of climate change.

Because of the uncertainties in the estimated costs, they should be taken as indicators of the size of the financial needs and not as accurate predictions. The estimates are likely to include both under- and over-estimates of the actual costs. Emerging technologies, along with significant investments in research and development, are likely to reduce current health burdens over the next 20+ years. On the other hand, the estimated costs were for only three of the health outcomes projected to increase with climate change; and then only a fraction of the burden of malnutrition was included. According to Caulfied et al. [11], the estimated prevalence of weight-for-age less than -2 SD (a measure of malnutrition) are 18% for Asia and the Pacific; 6% for Eastern Europe and Central Asia, and for Latin America and the Caribbean; 21% for the Middle East and North Africa; 46% in South Asia; 32% in Sub-Saharan Africa; and 2% in high-income countries. In addition, the model used to estimate malnutrition does not take into account new projections that a few degree increase in global mean temperature may render some areas unsuitable for rainfed agriculture; if this occurs, the short-term health consequences would likely be severe.

The costs estimated for adaptation are consistent with other estimates of financial needs for health care investment. Stenberg et al. [18] estimated the costs to scale-up essential child health interventions to reduce by two-thirds child mortality under the four MDGs aimed at children's health by 2015 in 75 countries; the countries chosen accounted for 94% of death among children less than five years of age. The interventions focused on malnutrition, pneumonia, diarrhoea, malaria, and key newborn causes of death. Calculations were bottom-up, based on intervention, country, and year. Costs included program-specific investments needed at national and district levels. The authors estimated that an additional US$ 52.4 billion would be required for the period 2006–2015. Projected costs in 2015 were equivalent to increasing the average total health expenditures from all financial resources in the 75 countries by 8% and raising general government health expenditure by 26% over 2002 levels. The authors noted that countries with weak health care systems may experience difficulties mobilizing enough domestic public funds.

Kiszewski et al. [19] estimated that US$ 38 to 45 billion would be required from 2006 to 2015 to scale up current malaria control programs to reach international goals, or about US$ 3.8 to 4.5 billion annually. If resources were to be made available and malaria goals were achieved, then the numbers of climate change-related cases of malaria in 2030 would likely to significantly lower, thus requiring fewer additional resources for treatment than the estimated US$ 4 – 12 billion under the 750 ppm CO_2 _scenario.

Although current governmental health expenditures can be anticipated to increase with development, there are health problems other than those associated with climate change that need to be addressed, such as HIV/AIDS, tuberculosis, diabetes, and other diseases. Assuming that Ministries of Health, NGOs, and other actors will completely cover the additional costs related to climate change is not realistic for many low-income countries; to do so would mean that other health issues of importance are left wanting. Financial and policy arrangements will need to be altered to address the projected additional cases of diarrhoeal diseases, malnutrition, and malaria.

## Conclusion

Overall, progress is being made in controlling climate-sensitive health outcomes. However, much of the progress has been in areas where the health outcomes are easier to control. The world is not on track to meet the health-related MDGs by 2015, with climate change working against disease control efforts.

Because the needs for investment in the health sector are large, capacity needs to be built to address climate-sensitive health outcomes. There needs to be increased awareness among Ministries of Health and donors of how climate change could alter the burden of a range of health outcomes, so that appropriate modifications are made in current programs to better address these health outcomes to increase future adaptive capacity. Additional human and financial resources will be needed to prevent and control the projected increased burden of health outcomes due to climate change.

## Abbreviations

CO_2:_ carbon dioxide; DALYs: Disability Adjusted Life Years; EMF: Energy Modelling Forum; MDGs: Millennium Development Goals; NGOs: Non-Governmental Organizations; ppm: parts per million;UE: unmitigated emissions.

## Competing interests

The author declares that she has no competing interests.

## Supplementary Material

Additional file 1 WHO regions. Countries within each WHO region, organized by mortality stratum. Click here for file

Additional file 2 Global Burden of Disease central and high projections of the relative risk of diarrhoea for alternative climate scenarios relative to baseline climate. Relative risks for diarrhoea in the year 2030 from the WHO Global Burden of Disease study under different climate scenarios. Click here for file

Additional file 3 Global Burden of Disease central and high projections of the relative risk of malnutrition for alternative climate scenarios relative to baseline climate. Relative risks for malnutrition in the year 2030 from the WHO Global Burden of Disease study under different climate scenarios. Click here for file

Additional file 4 Global Burden of Disease central and high projections of the relative risk of malaria for alternative climate scenarios relative to baseline climate. Relative risks for malaria in the year 2030 from the WHO Global Burden of Disease study under different climate scenarios. Click here for file
