# Supplementary material for: Adaptation costs for climate change-related cases of diarrhoeal disease, malnutrition, and malaria in 2030
Source: Global Health. 2008 Sep 19;4:9. doi: 10.1186/1744-8603-4-9 (PMC2556651; doi:10.1186/1744-8603-4-9)
Supplement: Additional file 1 — WHO regions. Countries within each WHO region, organized by mortality stratum. [file 1744-8603-4-9-S1.doc]

**Table 1: WHO r**egions

| Region and Mortality Stratum | Description | Broad Grouping | Member States |
| --- | --- | --- | --- |
| Africa | | | |
| Afr-D | Africa with high child and high adult mortality | High-mortality developing | Algeria, Angola, Benin, Burkina Faso, Cameroon, Cape Verde, Chad, Comoros, Equatorial Guinea, Gabon, Gambia, Ghana, Guinea, Guinea-Bissau, Liberia, Madagascar, Mali, Mauritania, Mauritius, Niger, Nigeria, San Tome and Principe, Senegal, Seychelles, Sierra Leone, Togo |
| Afr-E | Africa with high child and very high adult mortality | High-mortality developing | Botswana, Burundi, Central African Republic, Congo, Cote d’Ivoire, Democratic Republic of the Congo, Eritrea, Ethiopia, Kenya, Lesotho, Malawi, Mozambique, Namibia, Rwanda, South Africa, Swaziland, Uganda, United Republic of Tanzania, Zambia, Zimbabwe |
| Americas | | | |
| Amr-A | Americas with very low child and very low adult mortality | Developed | Canada, Cuba, United States of America |
| Amr-B | Americas with low child and low adult mortality | Low-mortality developing | Antigua and Barbuda, Argentina, Bahamas, Barbados, Belize, Brazil, Chile, Colombia, Costa Rica, Dominica, Dominican Republic, El Salvador, Grenada, Guyana, Honduras, Jamaica, Mexico, Panama, Paraguay, Saint Kitts and Nevis, Saint Lucia, Saint Vincent and the Grenadines, Suriname, Trinidad and Tobago, Uruguay, Venezuela (Bolivarian Republic of) |
| Amr-D | Americans with high child and high adult mortality | High-mortality developing | Bolivia, Ecuador, Guatemala, Haiti, Nicaragua, Peru |
| South-East Asia | | | |
| Sear-B | South-east Asia with low child and low adult mortality | Low-mortality developing | Indonesia, Sri Lanka, Thailand |
| Sear-D | South-east Asia with high child and high adult mortality | High-mortality developing | Bangladesh, Bhutan, Democratic People’s Republic of Korea, India, Maldives, Myanmar, Nepal, Timor-Leste |
| Europe | | | |
| Eur-A | Europe with very low child and very low adult mortality | Developed | Andorra, Austria, Belgium, Croatia, Cyprus, Czech Republic, Denmark, Finland, France, Germany, Greece, Iceland, Ireland, Israel, Italy, Luxembourg, Malta, Monaco, Netherlands, Norway, Portugal, San Marino, Slovenia, Spain, Sweden, Switzerland, United Kingdom |
| Eur-B | Europe with low child and low adult mortality | Developed | Albania, Armenia, Azerbaijan, Bosnia and Herzegovina, Bulgaria, Georgia, Kyrgyzstan, Poland, Romania, Serbia and Montenegro, Slovakia, Tajikistan, The former Yugoslav Republic of Macedonia, Turkey, Turkmenistan, Uzbekistan |
| Eur-C | Europe with low child and high adult mortality | Developed | Belarus, Estonia, Hungary, Kazakhstan, Latvia, Lithuania, Republic of Moldova, Russian Federation, Ukraine |
| Eastern Mediterranean | | | |
| Emr-B | Eastern Mediterranean with low child and low adult mortality | Low-mortality developing | Bahrain, Iran (Islamic Republic of), Jordan, Kuwait, Lebanon, Libyan Arab Jamahiriya, Oman, Qatar, Saudi Arabia, Syrian Arab Republic, Tunisia, United Arab Emirates |
| Emr-D | Eastern Mediterranean with high child and high adult mortality | High-mortality developing | Afghanistan, Djibouti, Egypt, Iraq, Morocco, Pakistan, Somalia, Sudan, Yemen |
| Western Pacific | | | |
| Wpr-A | Western Pacific with very low child and very low adult mortality | Developed | Australia, Brunei Darussalam, Japan, New Zealand, Singapore |
| Wpr-B | Western Pacific with low child and low adult mortality | Low-mortality developing | Cambodia, China, Cook Islands, Fiji, Kiribati, Lao People’s Democratic Republic, Malaysia, Marshall Islands, Micronesia (Federated States of), Mongolia, Nauru, Niue, Palau, Papua New Guinea, Philippines, Republic of Korea, Samoa, Solomon Islands, Tonga, Tuvalu, Vanuatu, Viet Nam |
