# Supplementary material for: Adaptation costs for climate change-related cases of diarrhoeal disease, malnutrition, and malaria in 2030
Source: Global Health. 2008 Sep 19;4:9. doi: 10.1186/1744-8603-4-9 (PMC2556651; doi:10.1186/1744-8603-4-9)
Supplement: Additional file 4 — Global Burden of Disease central and high projections of the relative risk of malaria for alternative climate scenarios relative to baseline climate. Relative risks for malaria in the year 2030 from the WHO Global Burden of Disease study under different climate scenarios. [file 1744-8603-4-9-S4.doc]

**Table 4: Global Burden of Disease central and high projections of the relative risk of *Plasmodium* *falciparum*** malaria for alternative climate scenarios relative to baseline climate

| **Sub-region** | **Climate** | **2000** | | **2010** | | **2020** | | **2030** | |
| --- | --- | --- | --- | --- | --- | --- | --- | --- | --- |
|  |  | Mid | High | Mid | High | Mid | High | Mid | High |
| Afr-D | S550 | 1.00 | 1.01 | 1.01 | 1.01 | 1.01 | 1.02 | 1.01 | 1.02 |
|  | S750 | 1.00 | 1.01 | 1.01 | 1.02 | 1.01 | 1.02 | 1.01 | 1.03 |
|  | UE | 1.01 | 1.01 | 1.01 | 1.03 | 1.02 | 1.04 | 1.02 | 1.05 |
| Afr-E | S550 | 1.02 | 1.04 | 1.04 | 1.08 | 1.06 | 1.12 | 1.07 | 1.15 |
|  | S750 | 1.02 | 1.05 | 1.05 | 1.10 | 1.07 | 1.15 | 1.09 | 1.18 |
|  | UE | 1.04 | 1.08 | 1.08 | 1.16 | 1.12 | 1.23 | 1.14 | 1.28 |
| Amr-A | S550 | 1.08 | 1.15 | 1.15 | 1.30 | 1.23 | 1.46 | 1.27 | 1.53 |
|  | S750 | 1.09 | 1.19 | 1.19 | 1.37 | 1.28 | 1.56 | 1.33 | 1.65 |
|  | UE | 1.15 | 1.26 | 1.29 | 1.59 | 1.44 | 1.88 | 1.51 | 2.03 |
| Amr-B | S550 | 1.02 | 1.04 | 1.04 | 1.09 | 1.07 | 1.13 | 1.08 | 1.16 |
|  | S750 | 1.03 | 1.05 | 1.05 | 1.11 | 1.08 | 1.16 | 1.10 | 1.19 |
|  | UE | 1.04 | 1.09 | 1.09 | 1.17 | 1.13 | 1.26 | 1.15 | 1.30 |
| Amr-D | S550 | 1.01 | 1.02 | 1.02 | 1.05 | 1.03 | 1.07 | 1.04 | 1.09 |
|  | S750 | 1.01 | 1.03 | 1.03 | 1.06 | 1.04 | 1.08 | 1.05 | 1.10 |
|  | UE | 1.02 | 1.04 | 1.04 | 1.09 | 1.07 | 1.13 | 1.08 | 1.17 |
| Emr-B | S550 | 1.00 | 1.00 | 1.00 | 1.00 | 1.00 | 1.00 | 1.00 | 1.00 |
|  | S750 | 1.00 | 1.00 | 1.00 | 1.00 | 1.00 | 1.00 | 1.00 | 1.00 |
|  | UE | 1.00 | 1.00 | 1.00 | 1.00 | 1.00 | 1.00 | 1.00 | 1.00 |
| Emr-D | S550 | 1.03 | 1.07 | 1.07 | 1.13 | 1.10 | 1.20 | 1.15 | 1.30 |
|  | S750 | 1.04 | 1.08 | 1.08 | 1.16 | 1.12 | 1.24 | 1.19 | 1.37 |
|  | UE | 1.06 | 1.13 | 1.13 | 1.26 | 1.19 | 1.38 | 1.29 | 1.59 |
| Eur-A | S550 | 1.00 | 1.00 | 1.00 | 1.00 | 1.00 | 1.00 | 1.00 | 1.00 |
|  | S750 | 1.00 | 1.00 | 1.00 | 1.00 | 1.00 | 1.00 | 1.00 | 1.00 |
|  | UE | 1.00 | 1.00 | 1.00 | 1.00 | 1.00 | 1.00 | 1.00 | 1.00 |
| Eur-B | S550 | 1.00 | 1.00 | 1.00 | 1.00 | 1.00 | 1.00 | 1.00 | 1.00 |
|  | S750 | 1.00 | 1.00 | 1.00 | 1.00 | 1.00 | 1.00 | 1.00 | 1.00 |
|  | UE | 1.00 | 1.00 | 1.00 | 1.00 | 1.00 | 1.00 | 1.00 | 1.00 |
| Eur-C | S550 | 1.07 | 1.13 | 1.13 | 1.27 | 1.00 | 1.20 | 1.25 | 1.50 |
|  | S750 | 1.08 | 1.16 | 1.16 | 1.33 | 1.00 | 1.25 | 1.31 | 1.61 |
|  | UE | 1.13 | 1.26 | 1.26 | 1.52 | 1.00 | 1.39 | 1.48 | 1.97 |
| Sear-B | S550 | 1.00 | 1.00 | 1.00 | 1.00 | 1.05 | 1.00 | 1.00 | 1.00 |
|  | S750 | 1.00 | 1.00 | 1.00 | 1.00 | 1.09 | 1.00 | 1.00 | 1.00 |
|  | UE | 1.00 | 1.00 | 1.00 | 1.00 | 1.00 | 1.00 | 1.00 | 1.00 |
| Sear-D | S550 | 1.00 | 1.00 | 1.00 | 1.00 | 1.00 | 1.00 | 1.00 | 1.01 |
|  | S750 | 1.00 | 1.00 | 1.00 | 1.00 | 1.00 | 1.00 | 1.01 | 1.01 |
|  | UE | 1.00 | 1.00 | 1.00 | 1.01 | 1.01 | 1.01 | 1.01 | 1.02 |
| Wpr-A | S550 | 1.07 | 1.14 | 1.14 | 1.28 | 1.21 | 1.42 | 1.25 | 1.49 |
|  | S750 | 1.09 | 1.17 | 1.17 | 1.34 | 1.26 | 1.52 | 1.30 | 1.60 |
|  | UE | 1.14 | 1.27 | 1.27 | 1.54 | 1.41 | 1.81 | 1.48 | 1.95 |
| Wpr-B | S550 | 1.06 | 1.12 | 1.12 | 1.25 | 1.18 | 1.37 | 1.22 | 1.43 |
|  | S750 | 1.08 | 1.15 | 1.15 | 1.30 | 1.23 | 1.45 | 1.26 | 1.53 |
|  | UE | 1.12 | 1.24 | 1.24 | 1.48 | 1.36 | 1.71 | 1.42 | 1.83 |
